# Autochthonous carbon loading of macroalgae stimulates benthic biological nitrogen fixation rates in shallow coastal marine sediments

**DOI:** 10.3389/fmicb.2023.1312843

**Published:** 2024-01-05

**Authors:** Yubin Raut, Casey R. Barr, Emily R. Paris, Bennett J. Kapili, Anne E. Dekas, Douglas G. Capone

**Affiliations:** ^1^Marine and Environmental Biology, University of Southern California, Los Angeles, CA, United States; ^2^Earth System Science, Stanford University, Stanford, CA, United States

**Keywords:** benthic N_2_ fixation, macroalgal decomposition, diazotroph community, coastal marine sediments, *nifH* amplicon sequencing

## Abstract

Macroalgae, commonly known as seaweed, are foundational species in coastal ecosystems and contribute significantly to coastal primary production globally. However, the impact of macroalgal decomposition on benthic biological nitrogen fixation (BNF) after deposition to the seafloor remains largely unexplored. In this study, we measure BNF rates at three different sites at the Big Fisherman's Cove on Santa Catalina Island, CA, USA, which is representative of globally distributed rocky bottom macroalgal habitats. Unamended BNF rates varied among sites (0.001–0.05 nmol N g^−1^ h ^−1^) and were generally within the lower end of previously reported ranges. We hypothesized that the differences in BNF between sites were linked to the availability of organic matter. Indeed, additions of glucose, a labile carbon source, resulted in 2–3 orders of magnitude stimulation of BNF rates in bottle incubations of sediment from all sites. To assess the impact of complex, autochthonous organic matter, we simulated macroalgal deposition and remineralization with additions of brown (i.e., *Macrocystis pyrifera* and *Dictyopteris*), green (i.e., *Codium fragile*), and red (i.e., *Asparagopsis taxiformis*) macroalgae. While brown and green macroalgal amendments resulted in 53- to 520-fold stimulation of BNF rates—comparable to the labile carbon addition—red alga was found to significantly inhibit BNF rates. Finally, we employed *nifH* sequencing to characterize the diazotrophic community associated with macroalgal decomposition. We observed a distinct community shift in potential diazotrophs from primarily *Gammaproteobacteria* in the early stages of remineralization to a community dominated by *Deltaproteobacteria* (e.g., sulfate reducers), *Bacteroidia*, and *Spirochaeta* toward the latter phase of decomposition of brown, green, and red macroalgae. Notably, the *nifH*-containing community associated with red macroalgal detritus was distinct from that of brown and green macroalgae. Our study suggests coastal benthic diazotrophs are limited by organic carbon and demonstrates a significant and phylum-specific effect of macroalgal loading on benthic microbial communities.

## Introduction

Nitrogen (N) is an important macronutrient that governs microbial community structure and is often considered to be limiting in many regions of the ocean (Zehr and Capone, 2020). Diazotrophs or N_2_ fixers are specialized prokaryotes that utilize the nitrogenase enzyme to carry out biological N fixation (BNF), the process of converting dinitrogen (N_2_) gas into a bioavailable source of N. These microorganisms have been well-investigated in open-ocean oligotrophic regions (Zehr and Capone, 2023). In comparison, benthic coastal ecosystems, which are considered to be eutrophic and areas of net N_2_ loss, are often under-surveyed for BNF (Fulweiler, 2023). However, numerous studies report BNF by an assortment of diazotrophic phylotypes in illuminated coastal habitats with different benthic primary producers such as photosynthetic microbial mats (Zehr et al., 1995; Paerl et al., 1996), seagrass (Capone, 1982; Welsh, 2000; Mohr et al., 2021), and salt marsh plants (Nedwell and Azni bin Abdul Aziz, 1980; Gandy and Yoch, 1988). More recently, there has been a growing body of evidence suggesting that benthic BNF is ubiquitous throughout global coastlines (Andersson et al., 2014; Hou et al., 2018; Jabir et al., 2018, 2021).

Notably, benthic macroalgae contribute significantly to global coastal primary production and create important coastal habitats (Duarte and Cebrián, 1996; Krause-Jensen and Duarte, 2016). There have also been many reports of diazotrophic macroalgal associations (DMAs) with living macroalgae (Head and Carpenter, 1975; Capone et al., 1977; Johnson et al., 2023). In recent years, there has been a major uptick in macroalgal blooms, often linked to anthropogenic nutrient loading, affecting coastal marine ecosystems, often with detrimental ecological impacts throughout the world (Lyons et al., 2014; Lapointe et al., 2018; Wang et al., 2020). Despite their global occurrence and potential to harbor diazotrophs, coastal macroalgal habitats and the underlying marine sediments remain largely under-investigated for BNF. Therefore, one major objective of this study is to investigate the prevalence of benthic BNF along a coastal rocky bottom habitat, i.e., Big Fisherman's Cove (BFC), which often house dense kelp forests, diverse macroalgae, and other macrophytes such as eelgrass (e.g., *Zostera*) beds (Tanner et al., 2019; Looby and Ginsburg, 2021; Ginsburg and Huang, 2022).

In many parts of the oligotrophic ocean (e.g., Pacific Ocean and S. Atlantic), iron, an important constituent of the nitrogenase metalloenzyme, often constrains diazotrophic biogeography (Sohm et al., 2011). In other regions of the ocean (e.g., N. Atlantic), phosphorous has been reported to limit diazotrophic activity (Sohm et al., 2008). Numerous studies have explored how similar nutrients (e.g., iron) impact diazotrophic communities in benthic ecosystems (Howarth et al., 1988; Yao et al., 2023). While carbon is rarely thought to limit photosynthetic diazotrophs inhabiting the euphotic zone, the availability of organic matter, ranging from simple carbon substrates to whale falls, has routinely been reported to enhance heterotrophic microbial activity, including BNF, in diverse marine sediments (Meyer-Reil, 1987; Bertics et al., 2010; Gier et al., 2016; Polyak et al., 2017; Dekas et al., 2018). In this study, we similarly expect the addition of labile organic substrates (i.e., glucose and macroalgal detritus) to stimulate benthic BNF rates in shallow coastal marine sediments.

Macroalgae tend to be carbon-rich, often with much higher carbon to N ratios (>15:1) than typical Redfield ratios observed with other phytodetritus such as phytoplankton (Duarte, 1992; Duarte and Cebrián, 1996). During macroalgal decomposition, the N pool can be depleted by the microbial community much faster than carbon, and accumulation of non-labile N substrates can result in N limitation (Tenore et al., 1979; Rice, 1982; Rice and Hanson, 1984; Raut et al., 2018). This provides an ecological advantage for heterotrophic diazotrophs which can readily supply their own N while tapping into the residual carbon pool of the macroalgal detritus.

In previous studies, relatively low BNF rates were associated with healthy macroalgal biomass at the beginning of long-term (16–32 days) decomposition experiments (Hamersley et al., 2015; Raut and Capone, 2021). There was a substantial increase in BNF rates following a few days (3–7) of decomposition that were likely fueled by leaching of labile carbon substrates from the macroalgal detritus and sustained for an intermediary time period (Hamersley et al., 2015; Raut and Capone, 2021). As the macroalgal detritus became more recalcitrant, nitrogenase activity declined significantly toward the latter stages of decomposition (Raut et al., 2018; Raut and Capone, 2021). Interestingly, labile carbon amendments throughout macroalgal remineralization were found to stimulate nitrogenase activity only at the beginning and terminal phases of decomposition. This suggests that rapid microbial degradation of macroalgae in the intermediate phase of decomposition can alleviate carbon limitation for the associated diazotrophic community (Raut et al., 2018; Raut and Capone, 2021).

A significant portion of macroalgal biomass undergoes burial and remineralization in the surrounding sediments of the macroalgal beds and can also be exported to deep sea sediments (Duarte and Cebrián, 1996; Dierssen et al., 2009; Krause-Jensen and Duarte, 2016). Currently, the impact of macroalgal deposition and decomposition on the benthic microbial community remains largely unknown. Therefore, another major objective of this study is to investigate the effects of macroalgal sedimentation and remineralization on benthic BNF rates in shallow coastal marine sediments. We employ acetylene reduction assays (ARAs) to measure BNF rates with sediment slurry incubations at the Wrigley Institute for Environment and Sustainability (WIES), adjacent to BFC. To assess the impact of labile carbon and autochthonous organic matter on benthic BNF rates, we carry out glucose (10 mM) additions and macroalgal amendment experiments using a variety of species across all three major clades of brown, green, and red seaweeds in parallel to the unamended sediment slurry incubations.

Sulfate-reducing bacteria are often found to be the major constituents of benthic diazotrophic communities (Capone, 1982; Gandy and Yoch, 1988; Jabir et al., 2021; Liesirova et al., 2023). Indeed, parallel benthic sulfate reduction and BNF rates further support their importance in shallow marine sediments (Bertics et al., 2010, 2013). In this study, we use additions of sodium molybdate, an inhibitor of sulfate-reducing bacteria, to assess the contribution of sulfate-reducing diazotrophs to benthic BNF at BFC. Interestingly, similar inhibition assays have been used to identify sulfate-reducing bacteria to be a major constituent of the diazotrophic community throughout macroalgal decomposition (Raut et al., 2018; Raut and Capone, 2021). However, there have been molecular surveys, typically focusing on the *nifH* gene, that have revealed a greater diversity of diazotrophs in coastal marine sediments (Jabir et al., 2018) and associated with macroalgal remineralization (Zhang et al., 2015; Aires et al., 2019). In this study, we leverage a similar metabarcoding approach with *nifH* sequencing to characterize the macroalgae-associated diazotrophic diversity and identify community shifts during the decomposition of several macroalgae typically found within the study site.

## Materials and methods

### Site description and sediment sampling

**Figure 1:**
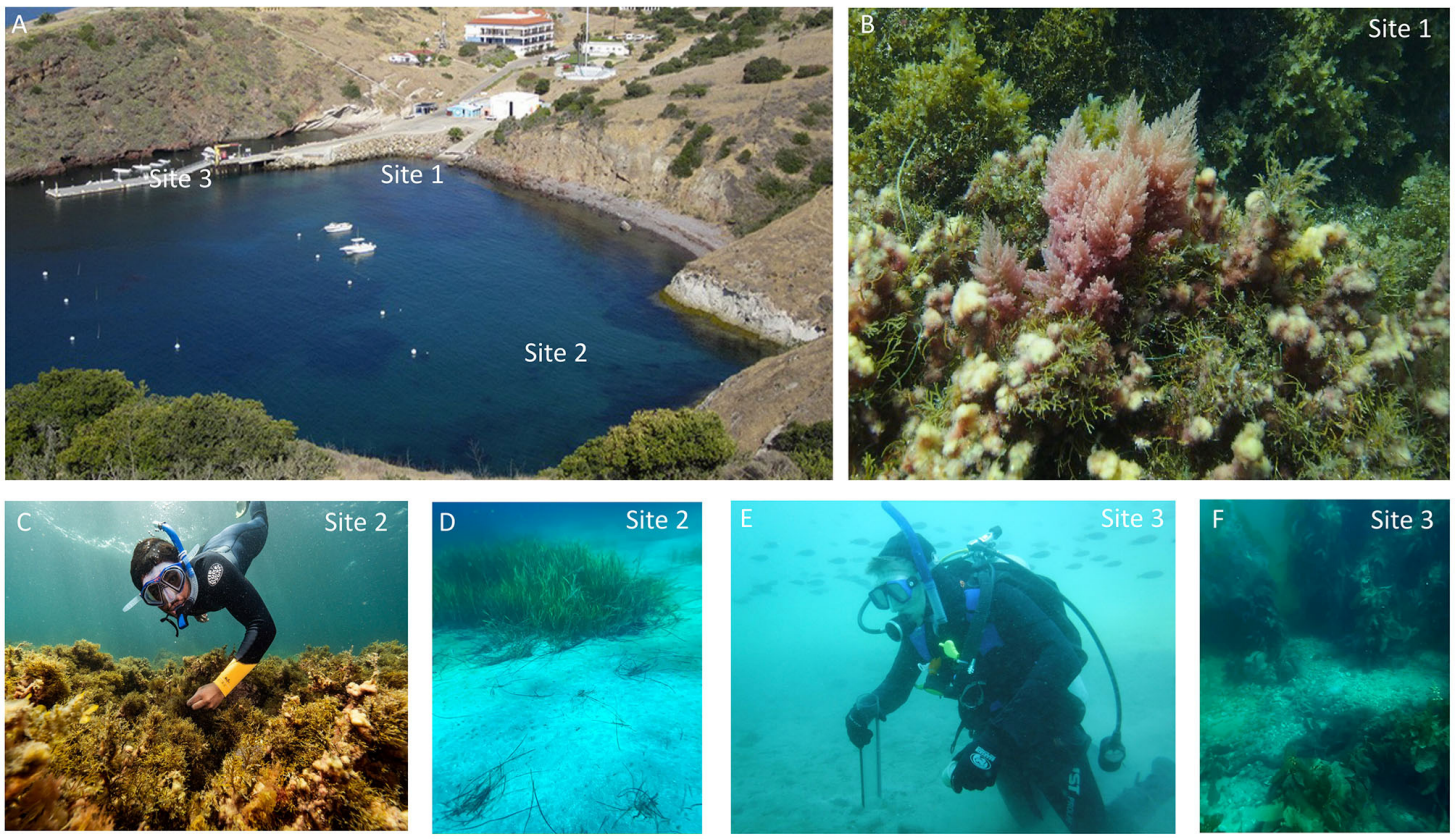
**(A)** Aerial view of Big Fisherman's Cove, Santa Catalina Island, California, USA depicting the three sites of survey for benthic BNF rates. **(B)** Site 1 (~3–5 feet water depth) is a more intertidal rocky zone populated by a mixture of macroalgae but often dominated by *Asparagopsis taxiformis*. **(C, D)** Site 2 (~10–15 feet water depth) is closer to the cliff wall where you might have terrigenous input and there's a stable population of eel grass (*Zostera*) and macroalgal assemblages of various brown and red macroalgae. **(E, F)** Site 3 is situated around the dock (~30 feet water depth) and populated by some giant kelp and steady aggregation of fish. Image credits: Yubin Raut—**(A)**, David W. Ginsburg—**(B)**, Maurice Roper—**(C)**, Jelani A. Williams—**(D, F)**, and Jasmine Henderson—**(E)**.

We surveyed three coastal marine sites for nitrogenase activity using the acetylene reduction assay (ARA) in the Big Fisherman's Cove (BFC), Santa Catalina Island, CA, USA, between spring and fall of 2017 (Figure 1A). The first location is a shallow, intertidal rocky site (~3–5 feet water depth) with mixed macroalgal assemblages consisting of various red (e.g., *Asparagopsis taxiformis*), green (e.g., *Codium fragile*), and brown (e.g., *Dictyopteris*) seaweed but primarily dominated by *A. taxiformis* (Figure 1B). The second location is a sandy bottom site (~10–15 feet water depth) situated adjacent to a stable eelgrass (*Zostera*) bed and shallow, rocky subtidal zone with mixed macroalgal assemblage (Figures 1C, D). The third sampling location is a sandy bottom site situated under a dock at an average water depth of ~30 feet with a low abundance of canopy-forming giant kelp (*Macrocystis pyrifera*) and a consistent aggregation of common Southern California fish. Site 3 is also adjacent to a rocky substratum housing many different fleshy brown macroalgae (Figures 1E, F). To investigate the role of autochthonous carbon sources on BNF rates at BFC, we carried out parallel incubations (experiments 2–4) with and without macroalgal amendments with sediments from the three sites. We measured the response of nitrogenase activity with the addition of brown (i.e., *M pyrifera* and *Dictyopteris*,), green (i.e., *C. fragile*), and red (i.e., *A. taxiformis*) macroalgae. These are commonly found at BFC (collected as healthy living seaweed at the time of sediment sampling) and along rocky bottom habitats all along the California coastline (Looby and Ginsburg, 2021; Ginsburg and Huang, 2022).

Sediment core samples were collected by hand on SCUBA using impact-resistant polycarbonate round tubes (3.5-inch OD, 3.25-inch ID x 1 ft, McMaster-Carr) and chemical-resistant tapered rubber plugs (McMaster-Carr). Duplicate cores were collected at each site, with an average depth of ~20 cm in May 2017. After collection, sediment cores were stored at 15°C in a constant temperature room at the adjacent WIES and subsampled for unamended BNF rate measurements representative of *in situ* benthic diazotrophic activity (experiment 1).

Additionally, ~25 L of sediment was also collected by hand on SCUBA at each site. Here, 5-gallon buckets were filled with material from the top 35 cm of the sediments, capped, and brought to the surface via a lift bag. To ensure the removal of both macrofauna and large sediment components (e.g., shells, rocks, and detritus), sediments were sieved through 500-μm copper mesh immediately upon collection. As a result of the sieving, sediment grains <500 μm were homogenized, disrupting any established geochemical gradients. Sieved materials were stored in 5-gallon buckets in the WIES 15°C room (similar to *in situ* water temperature), with aerated, UV-treated, 0.2 μm-filtered seawater being constantly pumped across each sediment collection (~20 L/day) to replenish oxygen levels within the overlying waters. The constant flow of filtered and UV-treated seawater prevents stagnation of the overlying waters and the removal of metabolic byproducts, creating a mesocosm mimicking that of the natural environment (Lam et al., 2019). The mesocosm established in May 2017 was subsequently sampled to conduct parallel unamended and amended sediment slurry incubations for BNF rate measurements in June and July 2017 (experiments 2 and 3). An additional mesocosm was freshly collected and set up in October 2017 which was subsequently sampled to conduct parallel unamended and amended sediment slurry incubations (experiment 4).

### Experimental design

There were a total of four separate experiments conducted where BNF rates were measured for a variety of unamended and amended sediment slurry incubations sampled from each of the three sites. **Experiment 1** consisted of unamended BNF rate measurements for sediment sampled from intact sediment cores collected in May 2017 and are representative of *in situ* benthic diazotrophic activity at the three sites. For **experiment 2** (June 2017), we subsampled from the initially established mesocosm (May 2017) to conduct parallel unamended and amended sediment slurry incubations. Here, we employed the following treatments: (1) unamended, (2) *M. pyrifera* additions, and (3) *M. pyrifera* + 20 mM sodium molybdate additions. For **experiment 3** (July 2017), we similarly subsampled from the same mesocosm (May 2017) to conduct another set of sediment slurry incubations consisting of (1) unamended treatment and (2) *A. taxiformis* additions. For **experiment 4** (October 2017), a new mesocosm was established and subsampled to conduct a final set of sediment slurry incubations with the following treatments: (1) unamended, (2) *C. fragile* additions, (3) *Dictyopteris* additions, (4) *M. pyrifera* additions, and (5) 10 mM glucose additions. All macroalgae used for the amendments were collected on the day of incubation and consisted of healthy, living tissue that was then either autoclaved or freeze-dried before being added to the incubation. Similarly, the glucose and sodium molybdate solutions were freshly made on the day of the incubation.

### Nitrogenase activity determined using the acetylene reduction assay (ARA)

All nitrogenase activity measurements for sediment slurry incubations were carried out in 27 mL serum vials flushed with N_2_ using the acetylene reduction assay (ARA) as detailed by Capone (1993) with some modifications. The volume of sediment sampled, using a 5-mL syringe, and the volume of fresh, 0.2 μM-filtered seawater added to the slurry incubations were 9 mL of sediment: 9 mL of seawater. For any of the macroalgal amendments, an additional ~0.5 g of biomass (autoclaved or freeze-dried living macroalgal tissue) was added to each vial. For the glucose and sodium molybdate amendments, a final concentration of 10 mM glucose and 20 mM sodium molybdate, respectively, was added in the 0.2-μM filtered seawater solution. In the remaining 9 mL of head space, 2 mL of acetylene gas (C_2_H_2_)—produced by reacting water with calcium carbide—was added with a gas-tight syringe resulting in a final volume of C_2_H_2_ (~7% of total volume or ~22% of the gas phase) that sufficiently saturated the nitrogenase enzyme (Flett et al., 1976). All ARAs were carried out in triplicate alongside a triplicate set of sediment slurry incubations without C_2_H_2_ additions that were monitored in parallel and showed no background ethylene (C_2_H_4_) production.

The vials were all incubated in the dark inside the same 15°C room as where the mesocosms were stored at WIES. We subsampled 100 μL of the headspace of each vial at different time points throughout the incubation and analyzed it on a Shimadzu Mini 2 gas chromatograph equipped with a flame ionization detector and coupled to a PeakSimple Chromatography Data System that was routinely calibrated at each time point with 100 ppm C_2_H_4_ standards to accurately quantify C_2_H_4_ peak heights. The initial time point consisted of a measurement at the time of C_2_H_2_ injection to establish a baseline of C_2_H_4_ production. Subsequently, we conducted time points every 6–15 h over the course of the ~5-day incubations, with increased monitoring during the intermediate period of incubations that excluded the lag and plateau in nitrogenase activity observed at the beginning and end, respectively, of incubations. It is important to note that ARA can have pronounced impacts on the sediment microbial community, including N_2_ fixers, sulfate reducers, and denitrifiers, in a relatively short time span (~7 h), and thus warrant careful interpretation of the results from longer incubations used in this study (Fulweiler et al., 2015). Furthermore, ARA can impact the physiology of the organisms, e.g., inhibition of sulfate-reducing bacteria (Dekas et al., 2009), which might result in under-estimation of total nitrogenase activity as it might inhibit some diazotrophic members of any community.

### Macroalgal litter bag decomposition

To observe potential shifts in the diazotrophic community over time, we sampled macroalgal detritus at multiple time points throughout separate long-term (16–32 days) decomposition experiments and sequenced the *nifH* genes of their associated microbial communities. The *nifH* gene encodes the essential iron protein subunit of nitrogenase, the enzyme that catalyzes BNF. This analysis was conducted separately from the benthic ARA incubations but included three of the four macroalgal species (i.e., *Dictyopteris, C. fragile*, and *A. taxiformis*) used in the sediment slurry incubations.

Representative macroalgae from each of the three clades (brown—*Dictyopteris*, green—*C. fragile*, and red—*A. taxiformis*) were separately collected between 2017 and 2019 at sites surrounding Santa Catalina Island, CA, USA, for monitoring DMAs throughout long-term macroalgal decomposition experiments as reported in Raut and Capone (2021). The freshly collected macroalgal biomass was divided into multiple individual 200 μM mesh litter bags, and a different bag was subsampled throughout the long-term decomposition of the macroalgae in flow-through seawater tanks at WIES. The different macroalgae were subsampled on the following days and immediately stored in −80°C freezers for downstream molecular work: *Dictyopteris* (2017—16-day decomposition experiment: days 0, 8, 10, and 14), *C. fragile* (2019—32-day decomposition experiment: days 0, 2, 4, 7, 10, 13, 17, 22, 26, and 32), and *A. taxiformis* (2017—18-day decomposition experiment: days 0, 5, 12, 15, and 18). The full nature of the long-term decomposition experimental setup is detailed in Raut and Capone (2021).

### DNA extraction, *nifH* gene amplification, and sequencing

Bulk community DNA extractions were done with macroalgal biomass subsampled on the previously specified days using the DNeasy PowerSoil Kit (Qiagen, Hilden, Germany, Cat. No. 12888-100). The following modifications were made to the quick-start protocol: (1) ~0.5 g of wet macroalgal biomass used, (2) samples vortexed for 20 min between moderate and high speeds during the initial bead beating step in the PowerBead tubes, and (3) centrifuged at 10,000 × *g* for 90 s during the initial supernatant collection. DNA concentrations were quantified using the protocol recommended with the Qubit 1X dsDNA HS Assay Kits (Invitrogen, USA, Cat. No. Q33230-Q33231).

The *nifH* gene was amplified in a two-step PCR process, with the first reaction containing: 1 μL DNA template, 12.5 μL Takara 2x Ex Taq Hot Start Premix (Takara Bio Inc., Kusatsu, Shiga, Japan), 0.5 μL 2.5 mg/mL bovine serum albumin (New England Biolabs, Ipswich, MA, USA), 0.5 μL each of forward and reverse *nifH* primers (10 μM; Mehta et al., 2003), and 10 μL sterile molecular-grade water. A touchdown PCR was conducted under the conditions reported in Supplementary Table 3 of Dekas et al. (2016). The second reaction was modified to include forward and reverse Illumina overhang adapters (5 μm) and was performed as follows: initial denaturation at 95°C for 120 s, followed by eight cycles of 95°C for 30 s, 55°C for 30 s, and 72°C for 45 s, and a final elongation step at 72°C for 5 min. A negative control (1 μL of molecular-grade water as template) was included for each forward and reverse Illumina barcode pair, and a positive control (various *nifH* sequences from a mock community) plus two randomly chosen sample replicates were run for each reverse Illumina barcode used. Barcoded PCR products were purified using the Ampure XP bead kit (Beckman Coulter) at 0.7 × bead solution, pooled in equal concentrations, purified again at 0.8 × bead solution, and sent to the UC Davis DNA Technologies Core Facility (UC Davis, CA, USA) for 2 × 250 bp paired-end sequencing on an Illumina MiSeq platform (as described in Kapili et al., 2020).

### Denoising and quality filtering *nifH* sequences

The *nifH* primer sequences were trimmed from demultiplexed fastq files using cutadapt (v.3.5; Martin, 2011), reads were quality filtered and pooled, chimeras were removed, and amplicon sequence variants (ASVs) were inferred using DADA2 (V1.12.1; Callahan et al., 2016). Further quality control included pruning ASVs that did not align to the *nifH* target region, and the remaining ASVs were incorporated into the reference alignment and tree using SEPP (v.4.4.0; Mirarab et al., 2012) as described by Kapili and Dekas (2021). Finally, we use the Phylogenetic Placement for Inferring Taxonomy (PPIT v.1.2.0), to interpret the *nifH* phylogenetic placement and infer the taxonomy of the ASVs (Kapili and Dekas, 2021). For the purpose of this study, ASVs identified as suspected *nifH* homologs were removed, and only ASVs with a taxonomic inference were used to assess the diazotrophic community.

### Data analysis and statistical methods

The data analysis and statistical methods described below were carried out using R (version 4.1.3). Following the modifications described by Raut et al. (2018), a linear regression model—excluding any lags and plateaus in nitrogenase activity at the beginning and end, respectively—of ARA incubations was fit to calculate rates of C_2_H_4_ production in nmol by converting peak heights of C_2_H_4_ measured throughout the ARA incubations (Capone, 1993; Breitbarth et al., 2004). Then, using a 3:1 ratio of C_2_H_2_ reduction:N_2_ reduction (Capone, 1993), theoretical BNF rates were estimated and multiplied by 2 to express as nmol N × g^−1^ (dw of sediment) × h^−1^ for all experiments.

Subsequently, to determine departures from normality for BNF rates, the Shapiro–Wilk test (“shapiro.test”) was used. To test for homogeneity of group variances in BNF rates, the Fligner-Killeen test (“fligner.test”), a non-parametric method based on ranks, was used. Since all experiments incorporated a two-factorial design, i.e., sites (three locations) and amendments (variable depending on the experiment), a two-way analysis of variance (ANOVA; “aov”) was performed, and if a significant *p*-value (< 0.05) was obtained, multiple pairwise comparisons were conducted between BNF rates of different groups using Tukey's honest significant difference (HSD) test (“TukeyHSD”). For multiple experiments, where the data distribution violated assumptions of normality and homoscedasticity, a non-parametric alternative of the two-way ANOVA (“t2way”) was carried out. For instances with a significant *p*-value (< 0.05), a *post-hoc* test similar to Tukey's HSD that conducts multiple pairwise comparisons using medians (“medpb”) was used to compare between independent groups of BNF rates (Wilcox, 2017). The non-parametric alternatives to the two-way ANOVA and Tukey's HSD are functions stored in the file “Rallfun-v40.txt” (January 2023) that is publicly available from https://osf.io/xhe8u/. The *p*-values from these comparisons that are statistically significant are defined in the different figures displaying BNF rates as follows: *p*-value < 0.001 (^***^), *p*-value < 0.01 (^**^), and *p*-value < 0.05 (^*^).

The Bray–Curtis dissimilarity metric was calculated using the % relative abundance of ASVs found in different macroalgae subsampled throughout long-term decomposition experiments using the “vegdist” function with default specifications. Non-metric multidimensional scaling (NMDS), which uses ranks orders of species and makes fewer assumptions of the dataset, was run using the Bray–Curtis distance matrix with the “metaMDS” function under some modifications from default settings (i.e., autotransform = F, wascores = T) to visualize the *nifH*-containing communities. A hierarchical cluster analysis was performed on the Bray–Curtis distances with the “hclust” function using the complete linkage method to identify similar clusters (k = 4). An analysis of similarities (ANOSIM) test was performed using the function “anosim” with default specifications for different factors of grouping observations (i.e., macroalgal species, hierarchical cluster, and stage of decomposition). A permutational multivariate analysis of variance (PERMANOVA) test was also performed using the function “Adonis” with default specifications for the previously specified factors of grouping observations. The “vegdist,” “metaMDS,” “anosim,” and “adonis” functions were all part of the vegan package (v.2.6-2).

## Results and discussion

### BNF rates at Big Fisherman's Cove (BFC) and the impact of labile carbon availability

**Figure 2:**
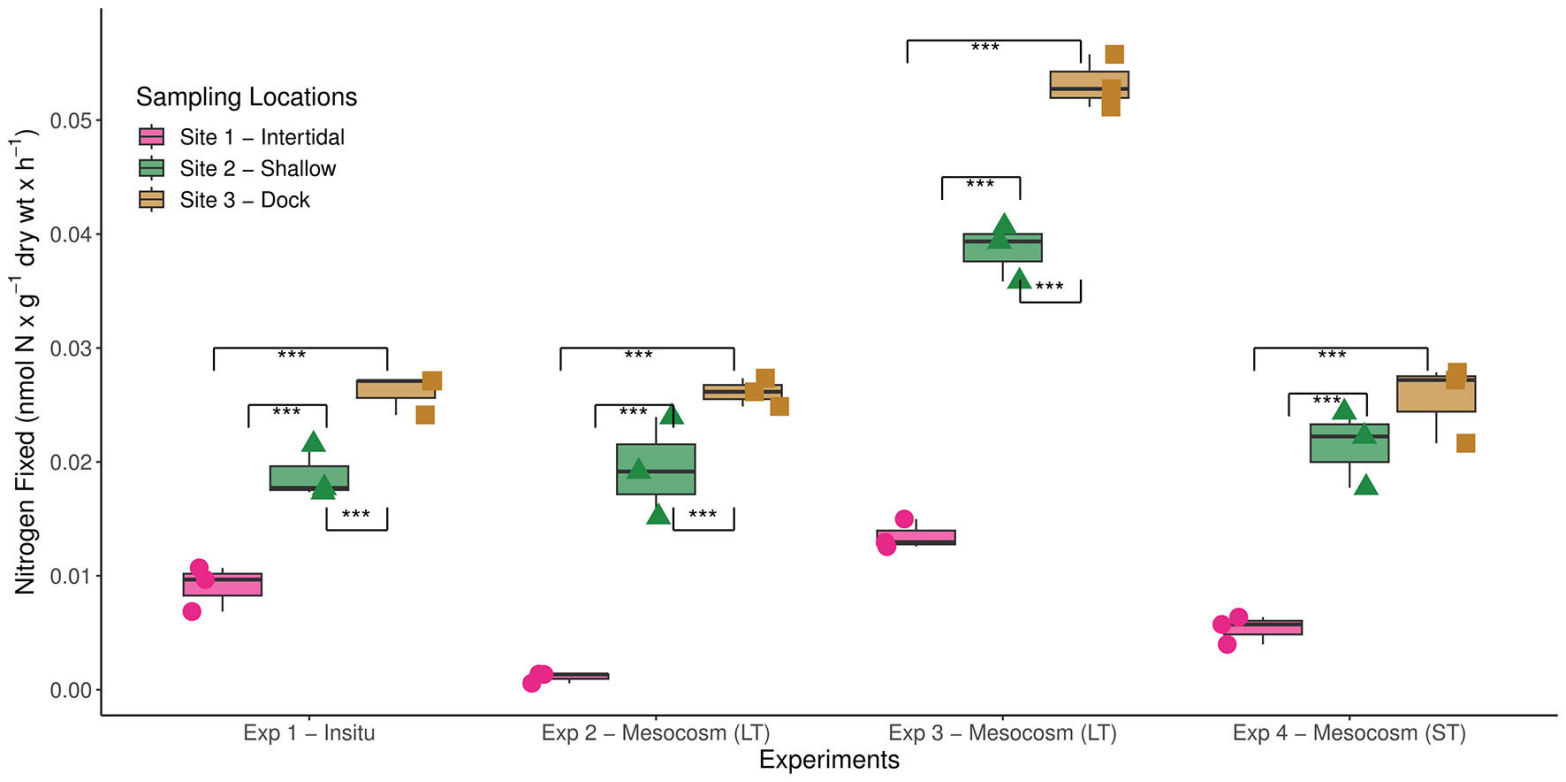
Biological nitrogen fixation (BNF) rates measured at the three different sampling sites in Big Fisherman's Cove, Santa Catalina Island, CA, USA during four separate experiments. The different experimental conditions are as follows: experiment 1—subsampled from intact sediment cores *in-situ* in May 2017, experiments 2 and 3—subsampled sediments from longterm (LT) mesocosms that were 1 (June 2017) and 2 (July 2017) months old, respectively, and experiment 4—established a new, short-term (ST) mesocosm (October 2017) that was subsampled shortly thereafter. The unamended BNF rates measured at each site during those four experiments are depicted in bar and whisker plots and statistically significant differences between sites are highlighted with brackets (***p-value < 0.001).

BNF rates measured at all three sites in BFC were within the range of previously reported BNF rates throughout a diverse array of coastal marine habitats, albeit at the lower end (Table 1). These lower rates could potentially be due to a deviation from the theoretical ratio (3:1) used to estimate BNF from ARA as observed in other systems (Soper et al., 2021) and could be refined in future studies by employing the more sensitive stable isotope tracer (e.g., 15 N_2_) methodology. Nonetheless, the diazotrophic activity was consistently higher at sites 2 and 3 in comparison with site 1 (Figure 2). We hypothesize that this could be due to greater sedimentation of metabolizable organic matter at sites 2 and 3 since benthic diazotrophic activity has been linked to organic matter availability previously (Capone and Budin, 1982; McGlathery et al., 1998). Additionally, potentially greater variability in environmental factors near the intertidal zone might also influence the lower BNF rates measured at site 1. For instance, increased wave exposure, as observed in this site, has previously been reported to have an overall negative impact on benthic diazotrophic activity across multiple shallow-water coastal sites (Andersson et al., 2014). Furthermore, the decreased availability of labile organic carbon can have another deleterious effect on the broader heterotrophic bacterial communities, including diazotrophs, at sites with greater wave exposure (Sander and Kalff, 1993).

**Table 1:** Compilation of benthic biological nitrogen fixation (BNF) rates from coastal marine sediments around the world.

**BNF rates (nmol N g^−1^ h^−1^)**	**Depth of integration (cm)**	**Environment and location**	**References**
0.001–0.05	0–20	Coastal inlet cove and marine protected area: Big Fisherman's Cove, Santa Catalina Island, CA, USA	This study—unamended incubations
1.1–4.1	0–20		This study—brown and green macroalgae (*M. pyrifera, Dictyopteris*, and *C. fragile*) amendments
4.7–5.7	0–20		This study—glucose (10 mM) amendments
0.005–0.03	0–20		This study—red macroalga (*A. taxiformis*) amendment
0.37–7.91	-	Intertidal estuarine sediments: Yangtze Estuary, China	Hou et al., 2018
0.27–5.67 (converted using 3:1)	0–5	Narrrangansett Bay, RI, USA	Spinette et al., 2019
0.1–1.11	-	Microtidal tropical estuary: Cochin, India	Jabir et al., 2021
0–0.2	10-cm intervals from 0–100	Mangrove sediments: Qi'ao Mangrove Wetland Park, Guangdong province, China.	Luo et al., 2021
0.013–10.199	0–5	Mariculture area: Muping Marine Ranch, Muping, Yantai, North Yellow Sea	Yao et al., 2023
**BNF rates (mmol N m**^−2^ **d**^−1^**)**	**Depth of integration (cm)**	**Environment and location**	**References**
0.8–8.5	0–10	Shallow, intertidal lagoon: Catalina Harbor, CA, USA	Bertics et al., 2010
0.008–0.22	0–18	Semi-enclosed bay: Ekernförde Bay, Germany	Bertics et al., 2013
0.03–1	0–1	Shallow (<1 m) coastal sediments: Sweden	Andersson et al., 2014
0.01–0.4	0–20	OMZ sediments: Peru	Gier et al., 2016
0.167–0.288	0–5	Coastal lagoon: San Quinfn Bay, Mexico	Hernández-López et al., 2017

The depth of integration for sediment incubations are reported when applicable and the marine environment and geographical location of the field sites are also reported for each study. The BNF rates are either reported in nmol N × g^−1^ (dry weight sediment) × h^−1^ or as mmol N × m^−2^ × d^−1^.

**Figure 3:**
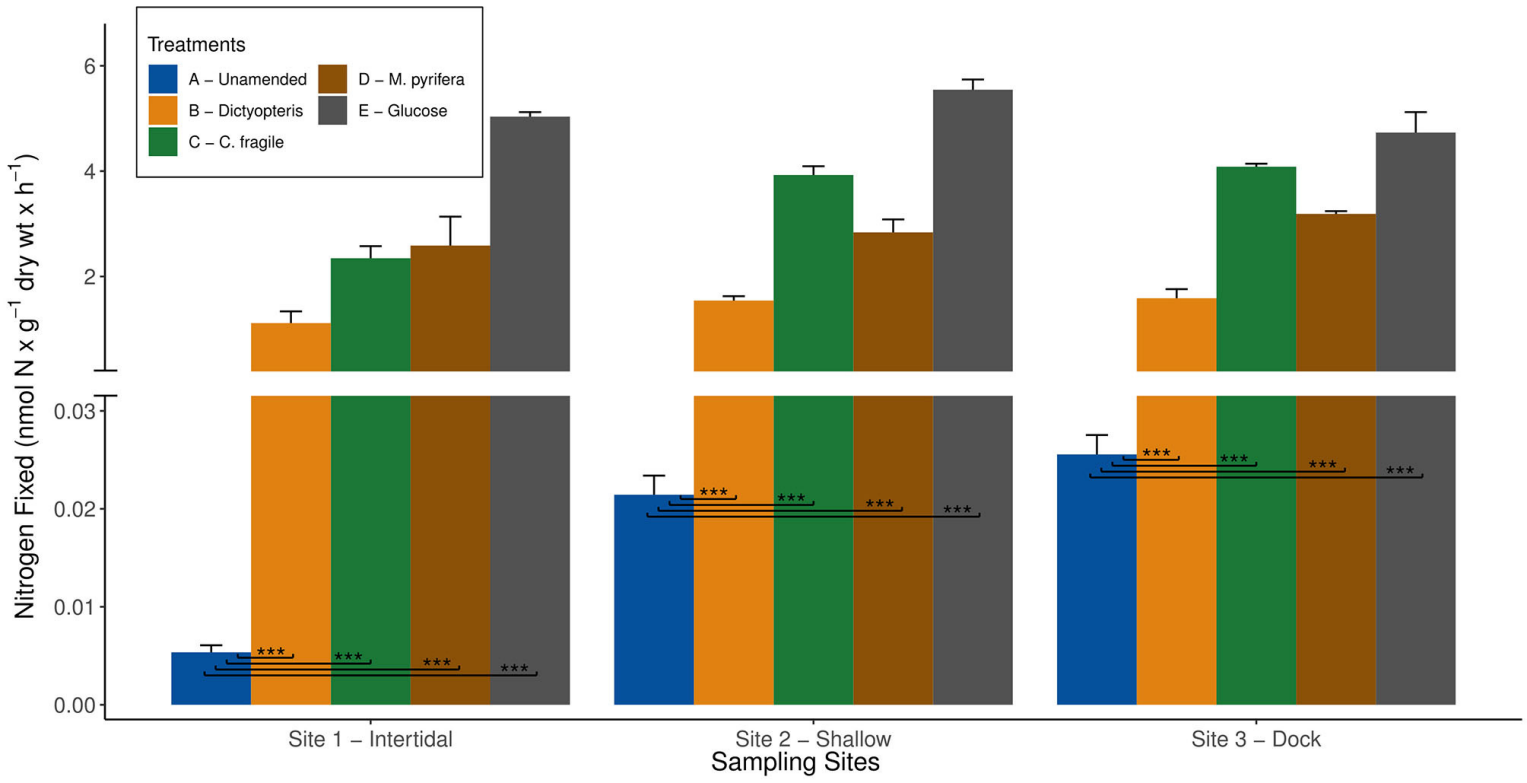
Average biological nitrogen fixation (BNF) rates (+/- standard error) measured at the three different sampling sites in Big Fisherman's Cove, Santa Catalina Island, CA, USA during experiment four in Fall 2017 are depicted by the bar plots. The blue bar represents unamended BNF rates, orange bar represents BNF rates with addition of brown macroalga *Dictyopteris*, green bar represents BNF rates with addition of green macroalga *Codium fragile*, brown bar represents BNF rates with addition of brown macroalga *Macrocystis pyrifera*, and gray bar represents BNF rates with addition of 10 mM glucose. Only the statistically significant difference between unamended (treatment A) and macroalgal and glucose amendments (treatments B - E) are depicted on the plot (***p-value < 0.001).

To test the potential for organic carbon to stimulate diazotroph activity, we conducted bottle incubations (experiment 4) with sediments from all three sites and added a labile carbon substrate. In previous studies, there have been a wide variety of carbon substrates (e.g., glucose, sucrose, and succinate) across a range of concentrations (1–10 mM) used to stimulate benthic BNF rates (Capone and Budin, 1982; McGlathery et al., 1998). Among the different carbon substrates tested, 10 mM glucose yielded the greatest stimulation (Capone and Budin, 1982), and therefore, the same concentration and substrate was selected in this study. Despite the baseline variability in unamended BNF rates among the three sites, the addition of glucose ubiquitously stimulated nitrogenase activity and resulted in comparable BNF rates between the sites (Figure 3). The pronounced 2–3 orders of magnitude increase in nitrogenase activity with 10 mM glucose addition compared to unamended BNF rates (*p*-value < 0.001) suggests that each site harbors a benthic diazotrophic community poised to respond to labile organic matter input (Figure 3). This highlights the BNF potential at each site, suggesting that the availability of labile organic matter can be a strong limiting factor for the benthic diazotrophic community at BFC. Furthermore, similar stimulation of nitrogenase activity with the addition of labile carbon substrates has been observed previously in many other benthic coastal habitats (Capone and Budin, 1982; McGlathery et al., 1998). The results from this study further support that labile carbon availability can strongly influence benthic diazotrophic activity.

### Role of autochthonous organic carbon on benthic diazotrophic community at BFC

**Figure 4:**
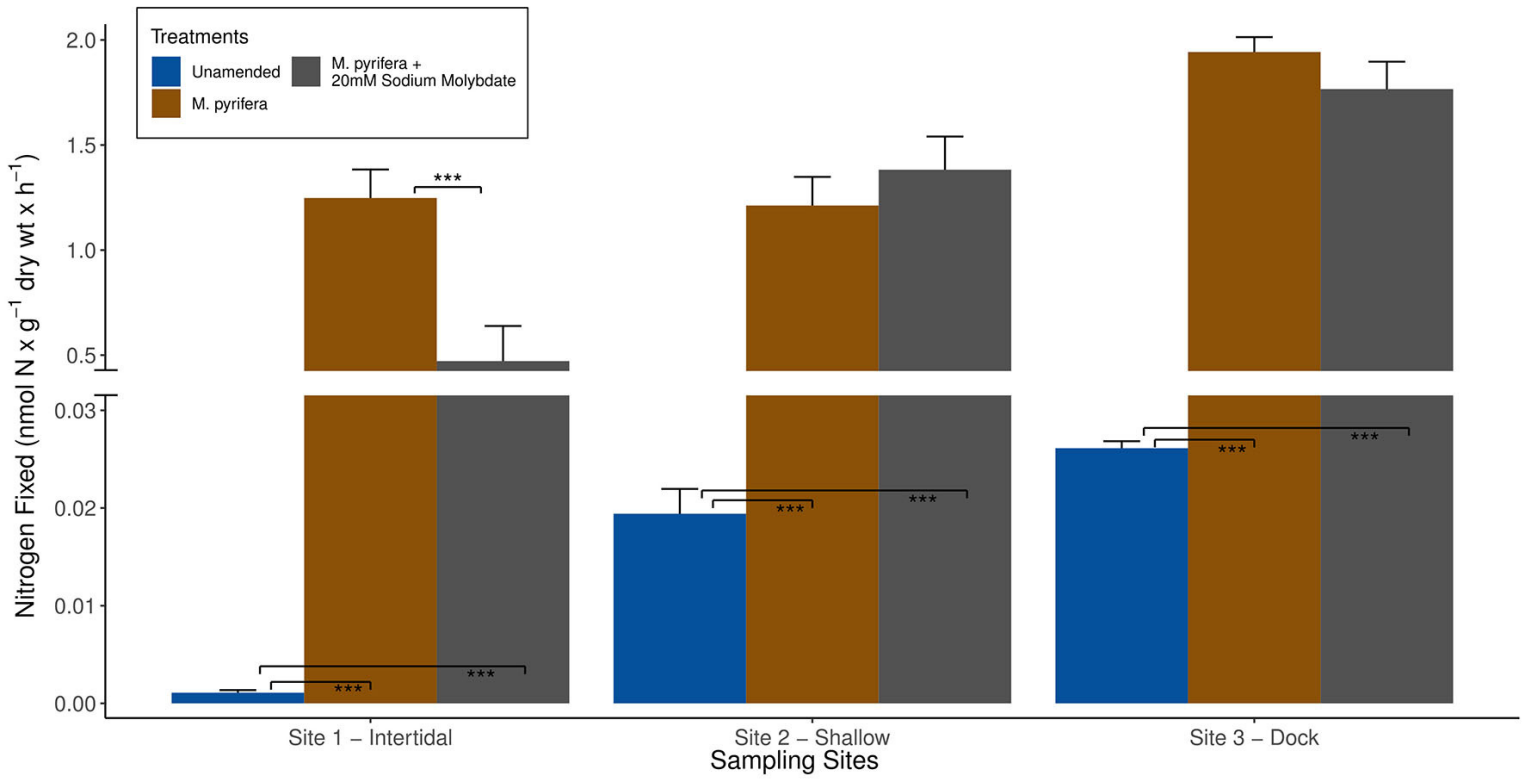
Average biological nitrogen fixation (BNF) rates (+/- standard error) measured at the three different sampling sites in Big Fisherman's Cove, Santa Catalina Island, CA, USA during experiment 2 (June 2017) are depicted by bar plots. The unamended BNF rates are in blue, BNF rates with addition of brown macroalga *Macrocystis pyrifera* in brown, and *M. pyrifera* plus 20 mM sodium molybdate, a strong inhibitor of sulfate reducers, additions are in gray. Only statistically significant differences are highlighted between the comparisons of (1) unamended incubation and two different amendments and (2) macroalgae only amendment vs. macroalgae + 20 mM sodium molybdate amendments: *p*-value < 0.001***.

In this study, we simulate the deposition of brown (i.e., *Dictyopteris* and *M. pyrifera*), green (i.e., *C. fragile*), and red (i.e., *A. taxiformis*) macroalgae by adding autoclaved or freeze-dried living macroalgal tissue to the sediment slurry incubations. Due to the length of ARA incubations (~5 days), there was sufficient time for the healthy macroalgal tissue to undergo remineralization, likely entering the intermediate stages of decomposition when the highest BNF rates are observed (Hamersley et al., 2015; Raut et al., 2018; Raut and Capone, 2021). Indeed, the brown and green macroalgal amendments (experiments 2 and 4) significantly stimulated BNF rates by ~2 orders of magnitude compared to unamended BNF rates (*p*-value < 0.001) across all three sites (Figures 3, 4). The remineralization of these brown and green macroalgae can release a substantial source of metabolizable carbon substrates, e.g., glucose, galactose, arabinose, mannitol, and laminarin (Zimmerman and Kremer, 1986; Ciancia et al., 2007), likely eliciting a similar microbial metabolic response as observed with the addition of 10 mM glucose (Figure 3).

Interestingly, the BNF rates between brown (i.e., *Dictyopteris* vs. *M. pyrifera*) and green (i.e., *C. fragile*) macroalgal amendments were notably different at sites 2 and 3 (*p*-value < 0.001; Figure 3). This potentially suggests that carbon loading of different macroalgal species, even within the same clade, might have different impacts on benthic diazotrophic activity (Figure 3). Furthermore, the ubiquitous response of nitrogenase activity across all three sites reinforces the notion that the benthic diazotrophic community is very strongly limited by the availability of labile organic matter. These results suggest that the autochthonous organic matter deposition of macroalgal biomass is an important source of labile carbon to the benthic diazotrophic community along coastal macroalgal habitats such as BFC (Figures 1, 3, 4).

**Figure 5:**
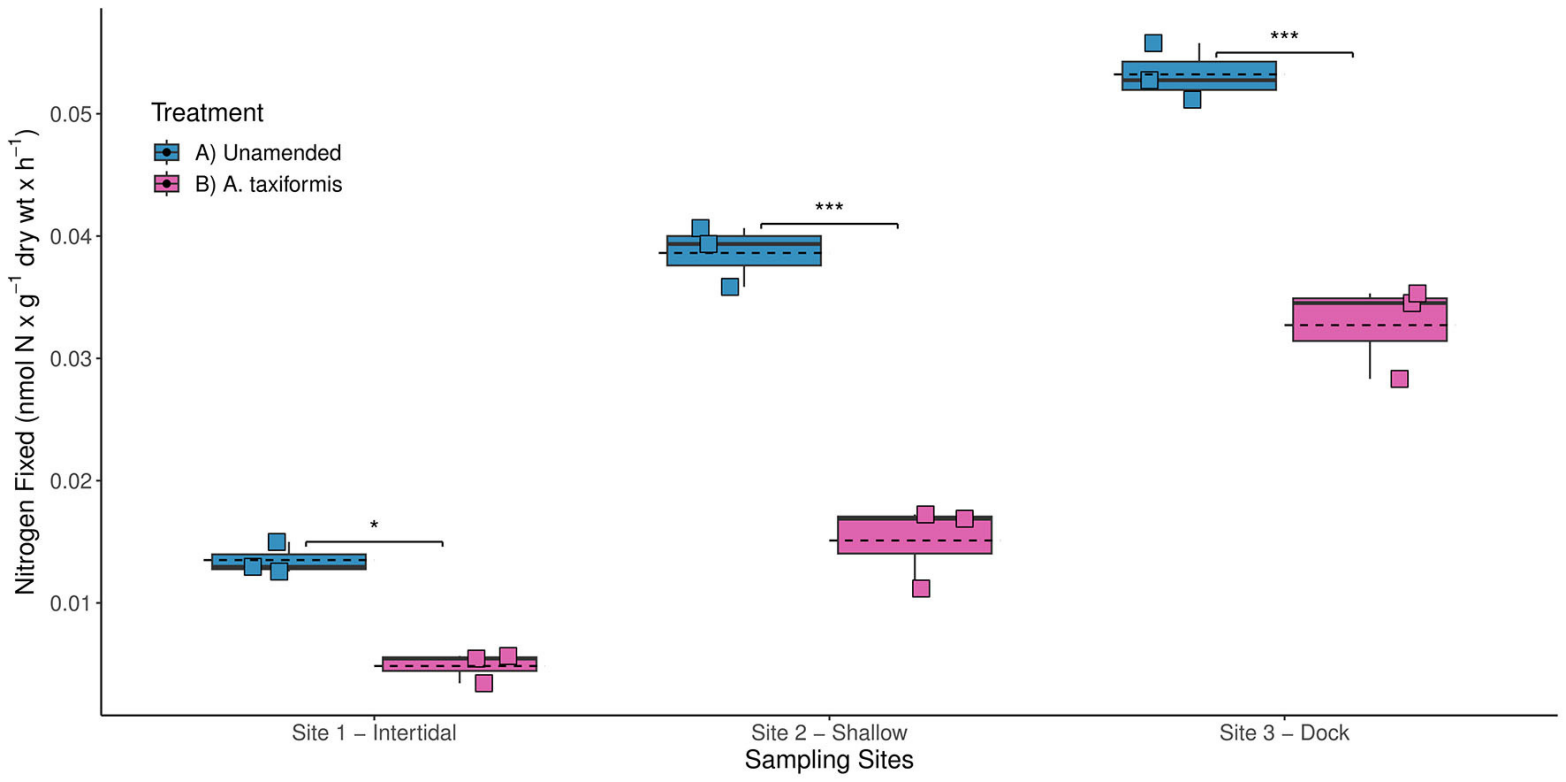
Biological nitrogen fixation (BNF) rates measured at the three different sampling sites in Big Fisherman's Cove, Santa Catalina Island, CA, USA during experiment 3 (July 2017). The unamended BNF rates (blue) and BNF rates with addition of red macroalga *Asparagopsis taxiformis* (magenta) are depicted using a box and whisker plot with dashed line representing the mean BNF rates for each treatment. Statistically significant values: *p*-value < 0.05*, *p*-value < 0.001***.

In previous studies, the highest BNF rates measured during the intermediate phase of decomposition of several red macroalgae (e.g., *Plocamium* and *A. taxiformis*) were ~2 orders of magnitude lower than BNF rates associated with brown and green macroalgae (Raut and Capone, 2021). Therefore, the addition of the red macroalga *A. taxiformis* was not expected to stimulate benthic BNF rates at any of the three sites. Surprisingly, the *A. taxiformis* amendments (experiment 3) were found to inhibit diazotrophic activity, yielding significantly lower BNF rates compared to the unamended BNF rates (*p*-value < 0.001) measured at all three sites (Figure 5). Reduced BNF rates could potentially be linked to the notable differences in stoichiometric ratios of carbon to N between *A. taxiformis* (<7) and other brown and green macroalgae (>10) (Raut and Capone, 2021). Here, the lower carbon to N content of *A. taxiformis* might have resulted in insufficient release of labile carbon to stimulate the benthic diazotrophic activity.

The release of halogenated secondary metabolites (e.g., bromophenol), which have strong antimicrobial activity (Mata et al., 2012), might have also prohibited microbial colonization during remineralization of *A. taxiformis* resulting in reduced diazotrophic activity. Additionally, *A. taxiformis* can be a significant source of phenolic compounds (Gao et al., 2022), which have been shown in other anoxic sediment systems, e.g., seagrass rhizosphere, to inhibit heterotrophic microbial consumption of sugars, e.g., sucrose (Sogin et al., 2022). Interestingly, the few microbes identified with the genes to degrade phenols and utilize sucrose within the seagrass rhizosphere were sulfate reducers and N_2_ fixers (Sogin et al., 2022). The decomposition of *A. taxiformis* could be selecting for fewer heterotrophic N_2_ fixers with the ability to degrade phenolic compounds and utilize the available sugars from the macroalgal detritus, resulting in lower bulk BNF rates than observed with remineralization of other macroalgae. Ultimately, this response by the diazotrophic community indicates that not all macroalgal deposition to the benthos will necessarily stimulate BNF rates.

Taken together, this suggests that the species composition of macroalgal sedimentation will play an important role in dictating the response by the benthic diazotrophic community. In fact, the increased prevalence of *A. taxiformis* throughout site 1 might further explain the consistently lower unamended BNF rates observed here in comparison with sites 2 and 3 (*p*-value < 0.001) in all four experiments (Figure 2). The chemical composition of the autochthonous source of carbon, e.g., macroalgal detritus, likely plays a significant role in determining the response by the benthic diazotrophic community to organic matter loading occurring along coastal marine habitats. Since significantly different BNF rates have been observed throughout the three stages of macroalgal decomposition (Hamersley et al., 2015; Raut et al., 2018; Raut and Capone, 2021), future studies should also explore how long-term macroalgal burial and remineralization in the marine sediment might impact benthic BNF rates. It is likely that the quality and lability of macroalgal detritus will influence benthic BNF rates, with the addition of more recalcitrant macroalgal detritus likely resulting in lower BNF rates compared to additions of healthier macroalgal tissue as used in this study.

### Diversity of macroalgae-associated diazotrophic community during decomposition

**Figure 6:**
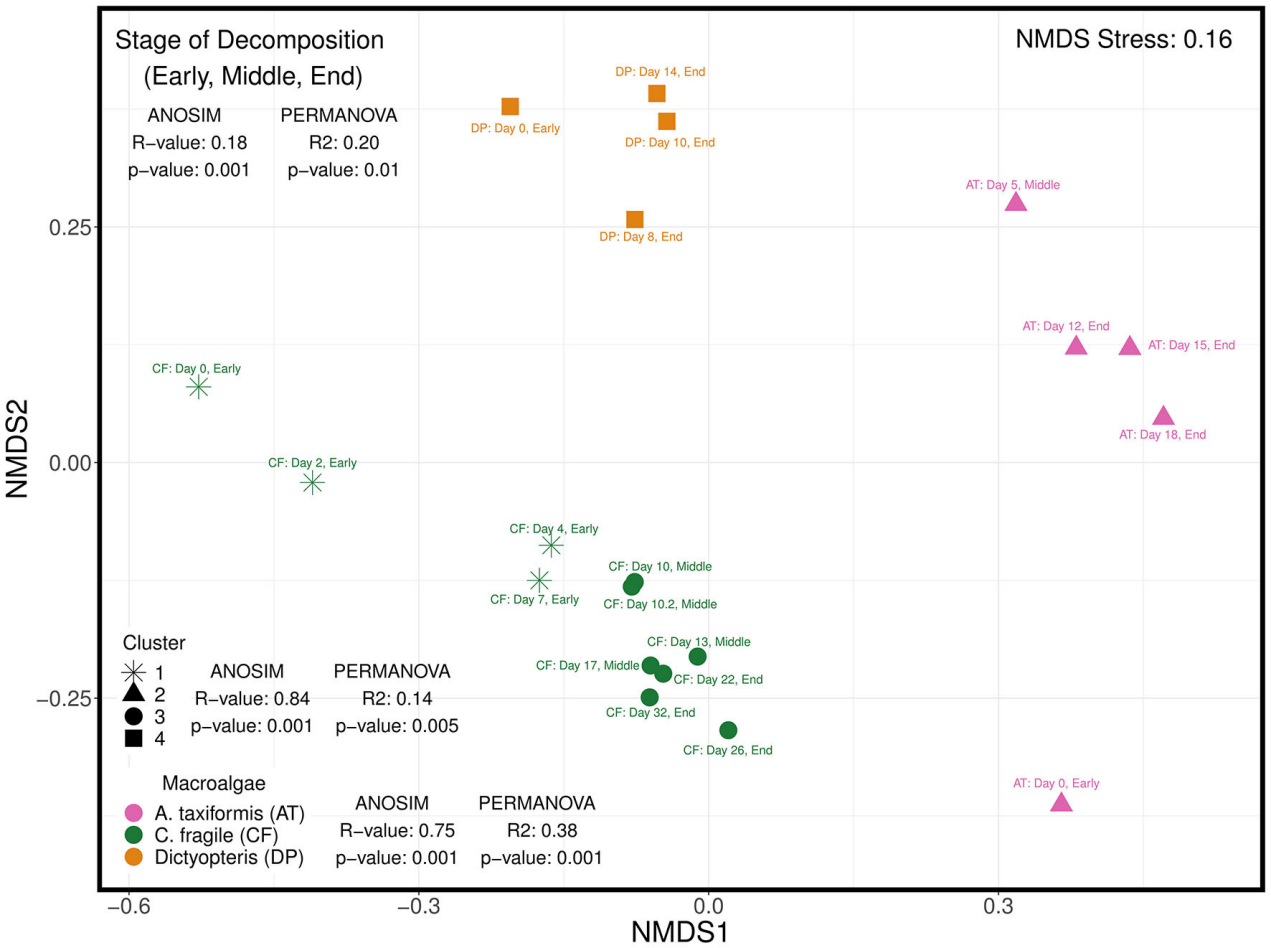
Non-metric dimensional scaling (NMDS) plot of Bray-Curtis dissimilarity indices of diazotrophic communities associated with different macroalgal species (*Asparagopsis taxiformis*—red macroalga; *Codium fragile*—green macroalga; *Dictyopteris*—brown macroalga) subsampled at different stages of decomposition: early, middle, and end. The stage of decomposition is denoted in the label next to each sample along with the exact day of sub-sampling from longterm litter bag decomposition. Hierarchical cluster analysis was also used to identify four major clusters denoted by the different shapes. The analysis of similarities (ANOSIM) test was used to determine how similar (closer to 0) or dissimilar (closer to 1) diazotrophic communities were to each other based on 3 different grouping variables: (1) macroalgal species composition, (2) hierarchical clusters, and (3) stage of decomposition of the macroalgal detritus. The permutational multivariate analysis of variance (PERMANOVA) test was used to further determine the percentage (*R*^2^ value) of variation explained by the 3 corresponding grouping variables.

The beta diversity of the diazotrophic communities was visualized on a non-metric multidimensional scaling (NMDS) plot using the Bray–Curtis dissimilarity indices and shows a clear distinction of diazotrophic communities associated with the different macroalgal species (Figure 6). An ANOSIM test was used to determine that the diazotrophic ASV assemblages were highly dissimilar between the three different species of macroalgae (*R*-value = 0.75, *p*-value = 0.001). A hierarchical cluster analysis was also used to identify four major clusters of samples (Figure 6), and an ANOSIM test similarly determined the diazotrophic assemblages to be highly dissimilar between the four clusters (*R*-value = 0.84, *p*-value = 0.001). A PERMANOVA test identified the hierarchical clusters (*R*^2^ = 0.14, *p*-value = 0.005) to explain a lower percentage of variation (14%) in diazotrophic communities across samples than macroalgal species composition (*R*^2^ = 0.38, *p*-value = 0.001, 38%; Figure 6).

Notably, there is an emerging feature with macroalgal samples where the diazotrophic community associated with the earlier stages of decomposition is distinct and partitions separately from samples in the intermediate and latter stages of decomposition (Figure 6). Indeed, categorical assignment of the three stages of decomposition, i.e., early, middle, and end, based on observation of the physical and structural integrity of each distinct macroalgal sample at the time of collection revealed that diazotrophic communities were very similar within each stage of decomposition (ANOSIM: *R*-value = 0.18, *p*-value = 0.001). The phase of decomposition was found to explain 20% (PERMANOVA: *R*^2^ = 0.20, *p*-value = 0.01) of the variation in diazotrophic communities across samples. Collectively, these observations suggest that the species composition and stage of decomposition of the macroalgal substrate likely play an important role in structuring the associated diazotrophic community.

**Figure 7:**
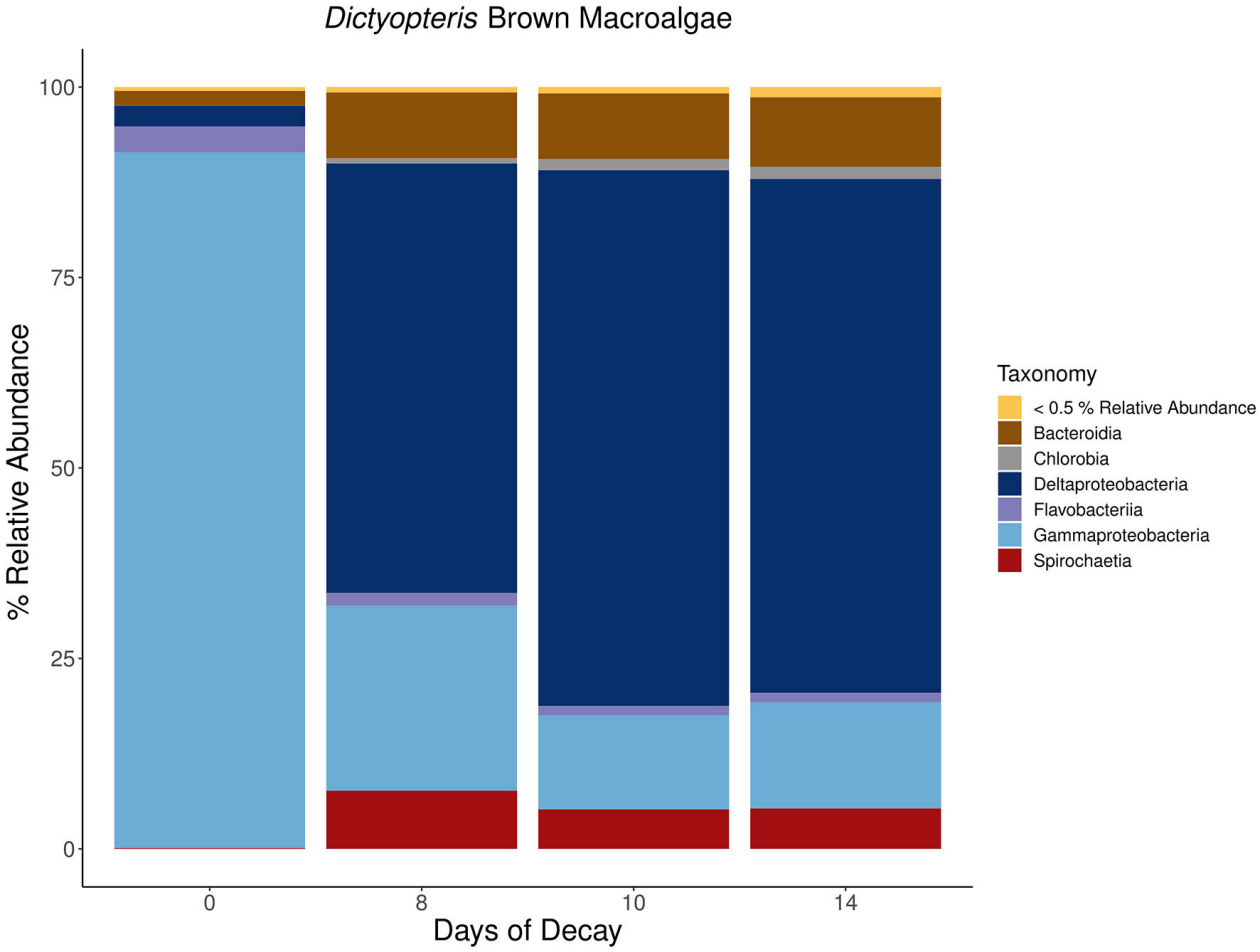
Stacked bar plots depicting the taxonomic composition of major *nifH*-containing amplicon sequence variants (ASVs) aggregated at the class or phylum level associated with macroalgal detritus sub-sampled on different days (i.e., 0, 8, 10, 14) throughout a long-term (16 days) litter bag decomposition of the brown macroalga, *Dictyopteris*. The remaining ASVs contributing <0.5% relative abundance of the total community per sample are aggregated together into a distinct group and can consist of a wide array of taxonomic diversity.

**Figure 8:**
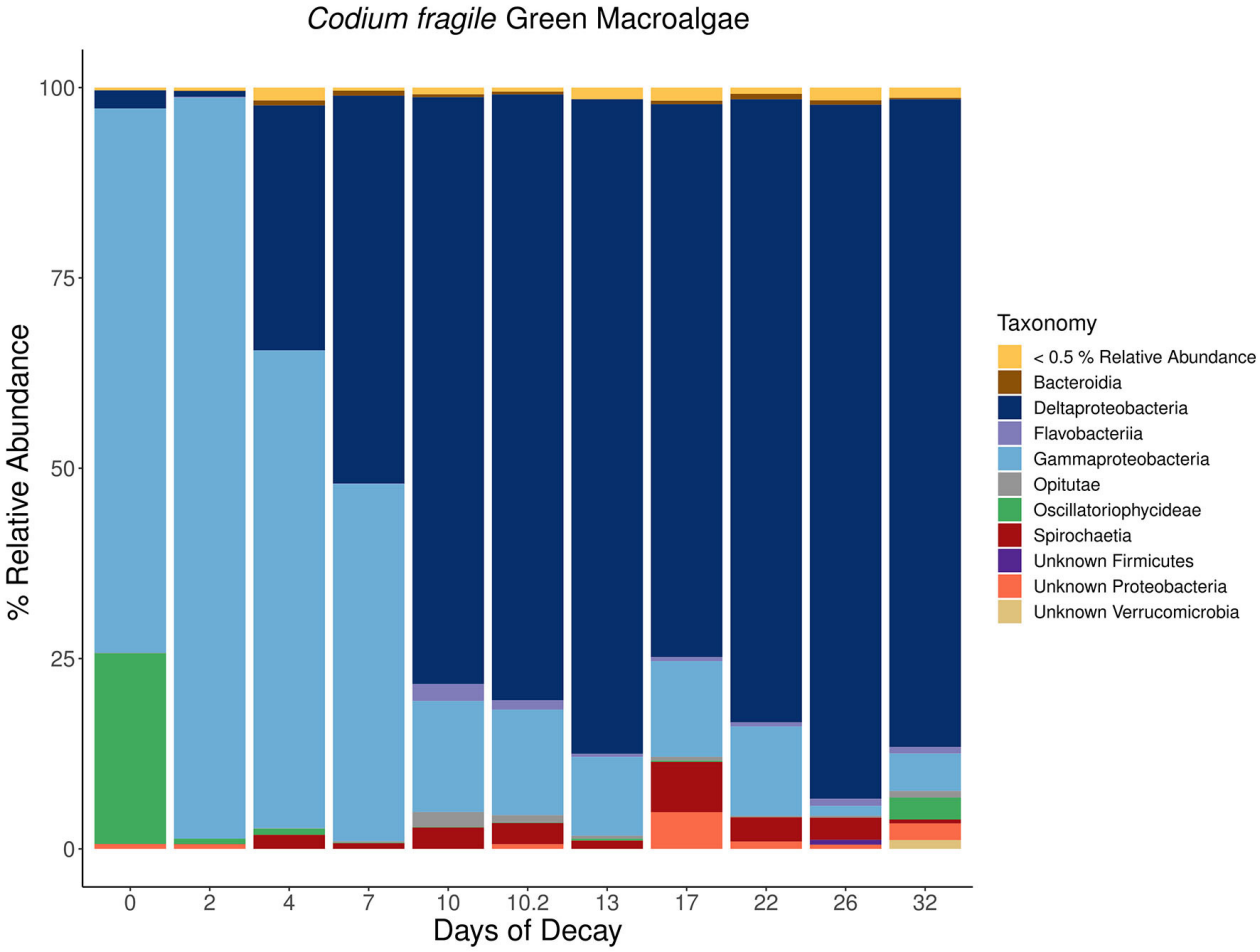
Stacked bar plots depicting the taxonomic composition of major *nifH*-containing amplicon sequence variants (ASVs) aggregated at the class or phylum level associated with macroalgal detritus sub-sampled on different days [i.e., 0, 2, 4, 7, 10 (technical sequencing replicate of day 10–10.2), 13, 17, 22, 26, 32] throughout a long-term (32 days) litter bag decomposition of the green macroalga, *Codium fragile*. The remaining ASVs contributing <0.5% relative abundance of the total community per sample are aggregated together into a distinct group and can consist of a wide array of taxonomic diversity.

**Figure 9:**
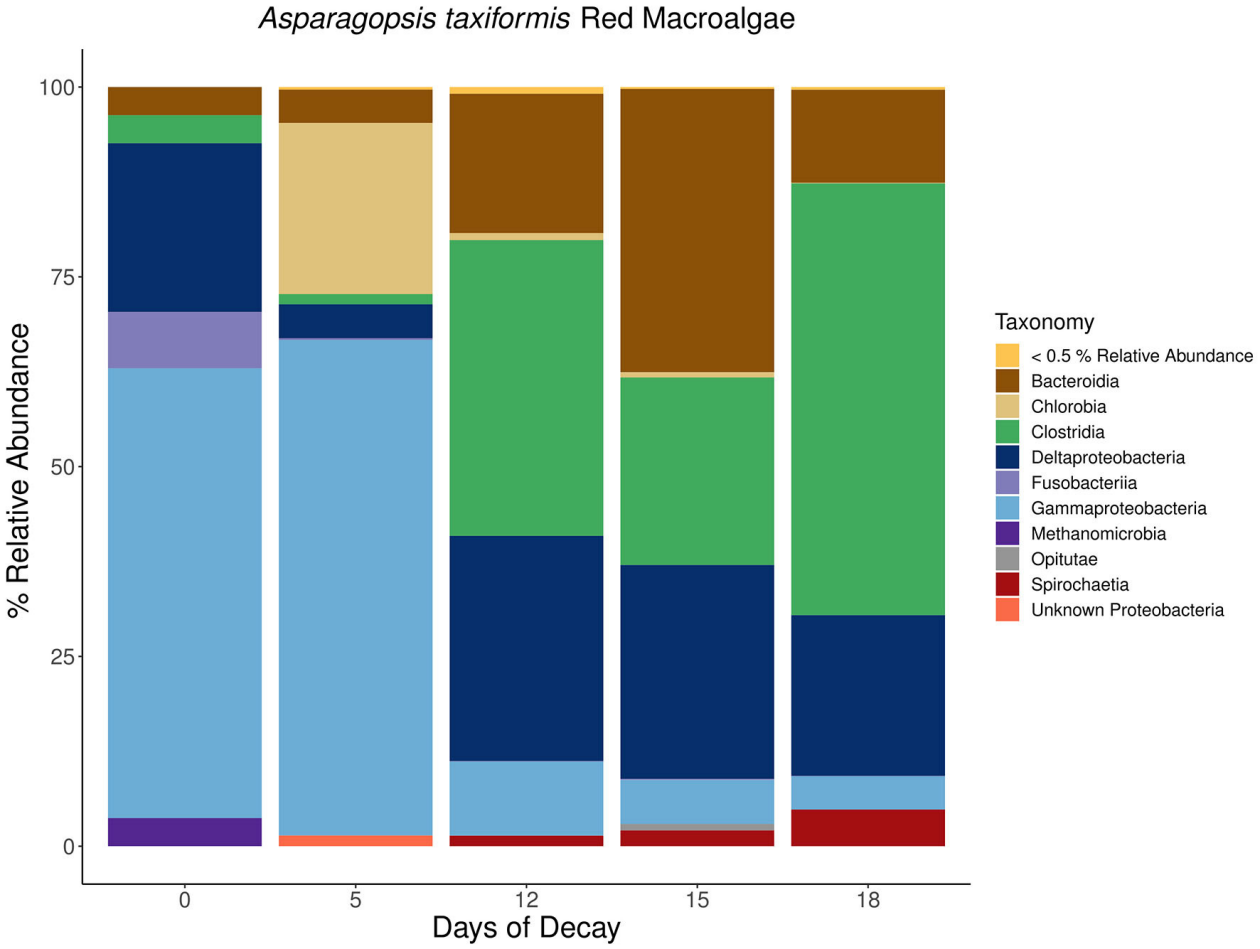
Stacked bar plots depicting the taxonomic composition of major *nifH*-containing amplicon sequence variants (ASVs) aggregated at the class or phylum level associated with macroalgal detritus sub-sampled on different days (i.e., 0, 5, 12, 15, 18) throughout a long-term (18 days) litter bag decomposition of the red macroalga, *Asparagopsis taxiformis*. The remaining ASVs contributing <0.5% relative abundance of the total community per sample are aggregated together into a distinct group and can consist of a wide array of taxonomic diversity.

For each macroalgal sample, microbial lineages inferred to host *nifH* that collectively contributed >0.5 % relative abundance were aggregated at the class or phylum level based on the inferred taxonomy using PPIT (Kapili and Dekas, 2021) and visualized in a stacked bar plot (Figures 7–9). The macroalgal-associated *nifH*-containing assemblages form distinct communities throughout the decomposition of brown, green, and red macroalgae, notably on the initial day of sampling where a higher abundance of cyanobacterial-*nifH* ASVs within *Oscillatoriophycideae* are observed (Figures 7–9). This is followed by a shift to *nifH* sequences associated with heterotrophic bacteria (Figure 8). This includes the ubiquitous presence of *nifH* sequences inferred to be from *Gammaproteobacteria* and *Deltaproteobacteria* (Figures 7–9).

For *C. fragile, Dictyopteris*, and *A. taxiformis*, the most prominent *nifH*-containing *Gammaproteobacteria* was from the genus *Vibrio*, where at times, a single *nifH* ASV could contribute up to 29, 13, and 19%, respectively, of the total *nifH*-containing community composition (Day 0). Furthermore, there were also ASVs with putative hosts within a wide array of orders including *Vibrionales, Alteromonadales, Aeromonadales, Methylococcales, Oceanospirillales, Thiotrichales*, and *Chromatiales* within the *Gammaproteobacteria* class. The most prevalent inferred *nifH* hosts within the *Deltaproteobacteria* were from the order *Desulfovibrionales* or *Desulfuromonadales*, with the most prevalent ASV contributing up to 57 (Day 26), 25 (Day 10), and 12% (Day 15) of the *nifH*-containing community composition associated with *C. fragile, Dictyopteris*, and *A. taxiformis*, respectively. There was also a wide array of ASVs from different families within the *Deltaproteobacteria* class including *Desulfovibrionaceae, Desulfuromonadaceae, Desulfobulbaceae, Desulfobacteraceae*, and *Geobacteraceae*. Similar diazotrophic members have also previously been observed with *nifH* surveys in coastal sediments (Jabir et al., 2018), including ecosystems populated by other macrophytes such as mangroves (Zhang et al., 2017) and seagrasses (Sun et al., 2015).

Collectively, there is a successional shift within the *nifH*-containing community from *Gammaproteobacteria*-dominated ASVs at the earlier stages of decomposition toward a more anaerobic assemblage of ASVs identified as from *Deltaproteobacteria, Spirochaeta*, and *Bacteroidia* in the intermediate and latter stages of remineralization (Figures 7–9). Members of the class *Spirochaetia*, including those within the genus *Spirochaeta*, are also inferred to host *nifH* sequences in these samples and are particularly prevalent during the intermediate and latter stages of macroalgal decomposition of all three brown, red, and green seaweeds (Figures 7–9). *Spirochete* bacteria, including N_2_ fixers, are often observed in anoxic aquatic environments, e.g., coastal marine habitats (Shivani et al., 2015), oxygen minimum zones (Loescher et al., 2014), and surface marine sediments (Jabir et al., 2021). After deposition onto marine sediments, it is likely that macroalgal decomposition fosters the development of anoxic conditions that would be favorable for similar *Spirochaetia* ASVs as those observed with macroalgal-associated detritus throughout long-term litter bag decomposition (Figures 7–9). There is also a prevalence of *Bacteroidia* associated with the brown macroalga, *Dictyopteris*, and the ASVs within this class consist of different families including *Prolixibacteraceae* and *Marinilabiliaceae* (Figure 7). Facultatively anaerobic *Draconibacterium* strains isolated from marine sediments (Du et al., 2014) and N_2_ fixing strains of *Mangrovibacterium* isolated from mangrove sediment (Huang et al., 2014) are both within the family *Prolixibacteraceae* which are the prominent *Bacteroidia* ASVs observed in this study. Furthermore, other facultatively anaerobic N_2_ fixing organisms within the *Bacteroidia* class have also been isolated from marine sediments with macrophyte (e.g., cordgrass and mangrove) presence (Huang et al., 2020).

Notably, the *nifH*-containing community associated with *A. taxiformis* was distinct from the community associated with the brown and green macroalgae, *Dictyopteris* and *C. fragile* (Figure 9). During the community succession throughout *A. taxiformis* remineralization, there is also a shift from *Gammaproteobacteria* during the earlier stages of decomposition to a *Deltaproteobacteria*-dominated *nifH*-containing community in the intermediate and latter stages. However, this transition is not marked by a homogenous shift toward a *Deltaproteobacteria* community but rather also consists of equal or greater contribution by *Bacteroidia* and *Clostridia nifH*-containing ASVs (Figure 9). Additional anaerobic fermentative *Firmicutes* species within the *Clostridia* class, including several representatives from the families *Defluviitaleaceae, Lachnospiraceae*, and *Clostridiaceae*, were also observed to be in high abundance toward the latter stages of *A. taxiformis* degradation (Figure 9). These findings suggest that while *Gammaproteobacteria* and *Deltaproteobacteria* are the dominant members of the macroalgal-associated *nifH*-containing communities, they may also be accompanied by a more diverse assemblage of *Spirochaeta, Bacteroidia*, and *Clostridia* N_2_ fixers (Figures 7–9).

Since *nifH* sequences inferred to be from *Gammaproteobacteria* were observed throughout the earlier (<5 days) stage of macroalgal decomposition (Figures 7–9), we hypothesize that macroalgal additions during the ~5-day ARA of sediment slurry incubations might stimulate the resident *Gammaproteobacteria* members of the benthic diazotrophic community. In this study, the shorter incubation and remineralization time period would likely not provide sufficient time for the succession to a *Deltaproteobacteria*-dominated community consisting of sulfate-reducing phylotypes (Jabir et al., 2021). Consistent with this, incubations amended with a macroalgal carbon source and 20 mM sodium molybdate, a potent inhibitor of sulfate reduction, only resulted in a significant reduction of BNF rates (*p*-value < 0.001) compared to incubations with only the macroalgal carbon source at site 1 (Figure 4). We hypothesize that this might be due to an existing difference in benthic diazotrophic assemblages between the three sites, perhaps with greater sulfate reducers present at site 1. In the future, *nifH* sequencing of marine sediment from the different sites could be helpful in characterizing *nifH*-containing community and deconvoluting potential differences in the microbial community between sites. Additionally, a longer time series could be used to directly monitor the succession of benthic diazotrophic assemblages throughout macroalgal remineralization, especially to investigate whether a similar shift from *Gammaproteobacteria* toward a *Deltaproteobacteria-*dominated community occurs as observed with the macroalgal-associated *nifH*-containing community.

## Conclusion

Conventionally, BNF is often thought to occur where N is limiting and thus is often under-surveyed in coastal marine sediments, especially macroalgal ecosystems where high macroalgal productivity (e.g., bloom events) could serve as an important source of organic matter. This study reevaluates that paradigm using biogeochemical measurements concurrently with molecular tools to disentangle the systematic impact that remineralization of macroalgae has on benthic BNF rates and characterizes the *nifH*-containing microbes driving this process in coastal marine ecosystems. Our study suggests coastal benthic diazotrophs are limited by organic carbon and demonstrates a significant and phylum-specific effect of macroalgal loading on benthic microbial communities.

## Data availability statement

The original contributions presented in the study are deposited in the European Nucleotide Archive (ENA) repository under accession number PRJEB71000 (secondary accession: ERP155889).

## Ethics statement

Written informed consent was obtained from the individual(s) for the publication of any potentially identifiable images or data included in this article.

## Author contributions

YR: Conceptualization, Data curation, Formal analysis, Funding acquisition, Investigation, Methodology, Visualization, Writing—original draft, Writing—review & editing. CRB: Conceptualization, Investigation, Methodology, Writing—review & editing. ERP: Data curation, Formal analysis, Methodology, Writing—review & editing. BJK: Data curation, Formal analysis, Methodology, Software, Writing—review & editing. AED: Funding acquisition, Resources, Supervision, Writing—review & editing. DGC: Funding acquisition, Resources, Supervision, Writing—review & editing.
